# Ethical aspects of medical age assessment in the asylum process: a Swedish perspective

**DOI:** 10.1007/s00414-017-1730-3

**Published:** 2017-11-11

**Authors:** Erik Malmqvist, Elisabeth Furberg, Lars Sandman

**Affiliations:** 1 Department of Culture and Communication, Linköping University, 581 83 Linköping, Sweden; 2 Centre for Research Ethics and Bioethics, Department of Public Health and Caring Sciences, Uppsala University, Uppsala, Sweden; 3 National Center for Priority Setting in Health-Care, Department of Medicine and Health, Linköping University, Linköping, Sweden; 4 Academy of Care, Welfare and Work-Life, University of Borås, Borås, Sweden

**Keywords:** Age assessment, Asylum seekers, Ethical issues, Unaccompanied minors

## Abstract

According to European regulations and the legislations of individual member states, children who seek asylum have a different set of rights than adults in a similar position. To protect these rights and ensure rule of law, migration authorities are commonly required to assess the age of asylum seekers who lack reliable documentation, including through various medical methods. However, many healthcare professionals and other commentators consider medical age assessment to be ethically problematic. This paper presents a simplified and amended account of the main findings of a recent ethical analysis of medical age assessment in the asylum process commissioned by the Swedish National Board of Health and Welfare. A number of ethical challenges related to conflicting goals, equality and fairness, autonomy and informed consent, privacy and integrity, and professional values and roles are identified and analysed. It is concluded that most of these challenges can be met, but that this requires a system where the assessment is sufficiently accurate and where adequate safeguards are in place. Two important ethical questions are found to warrant further analysis. The first is whether asylum seekers’ consent to the procedure can be considered genuinely voluntary. The second is whether and how medical age assessments could affect negative public attitudes towards asylum seekers or discriminatory societal views more generally.

## Introduction

According to European regulations and the legislations of individual member states, children who seek asylum have a different set of rights than adults in a similar position [1]. However, many asylum seekers lack documentation that certifies their age. This has prompted migration authorities to assess the age of asylum seekers in different ways, including through various medical methods [2]. In 2015, the Swedish National Board of Health and Welfare (*Socialstyrelsen*) initiated a revision of their previous recommendations for medical age assessment of unaccompanied minors who seek asylum in Sweden [3]. As a part of this work, the authors of the present paper were commissioned to analyse the ethical issues that such assessments raise. The analysis resulted in a report in Swedish [4]. The purpose of the present paper is to present our main findings and the reasoning that underlies them to a wider international audience. This, we hope, will contribute insights to the ongoing international debate on the ethics of age assessments in the asylum process [5–8] and complement recent reports about such measures from other countries [8, 9].

## Background

The Swedish National Board of Health and Welfare’s decision to revise its recommendations for medical age assessments had been preceded by rapidly increasing numbers of unaccompanied minors seeking asylum in Sweden [10] as well as by a consecutively polarised political and public debate about migration policy. Moreover, several Swedish paediatricians and scholars publicly took a stance against medical age assessments, mainly on the grounds that available methods were unacceptably inaccurate [11, 12]. At the time when the revision was initiated, few such assessments were being performed [13]. The controversy in Sweden has been paralleled by a heated international debate. Several European organisations for healthcare professionals have recommended their members not to participate in medical age assessments or urged that medical methods be combined with psychosocial methods in a holistic manner [14].

According to the Swedish regulatory framework, the age of an asylum seeker matters in two ways. First, it affects the individual’s rights during the asylum process. Unlike adults, minors who seek asylum are entitled to school attendance, full access to health and dental care, and (when unaccompanied by their legal guardian) special secure housing and a custodian who promotes their interest. Second, age can be of relevance to the outcome of the asylum process. According to the Swedish Aliens Act (2005: 716) Chapter 1 §10, decisions concerning children should be made with special consideration for their health and development as well as their best interests more generally. In a Government Bill (Prop 2004/05:170), this is interpreted to imply that the circumstances that motivate granting asylum need not be as severe in the case of children as in the case of adults. Also, in keeping with EU Regulation No. 604/2013, children may apply for asylum in Sweden regardless of where they entered the EU whereas adults must file the application in the country where they first arrived.

Medical age assessment is not a routine part of the Swedish asylum process. Rather, reflecting Directive 2013/32/EU Art. 25(5), such an assessment becomes relevant in cases where the Migration Agency (*Migrationsverket*) questions the stated age of an asylum seeker and the latter cannot support the statement with reliable documentation. In such a situation, the onus is on the asylum seeker to provide evidence for their stated age. Such evidence could be drawn from medical age assessments or from other sources. The Migration Agency has a duty to inform asylum seekers whose minority status is questioned of the possibility of requesting an age assessment and to fund the assessment if they do request one. However, the Agency does not itself perform age assessments, nor can it require asylum seekers to undergo them. Rather, the procedure takes place on the initiative of the asylum seeker.

## Limitations and points of departure

In order to provide a focused analysis, we had to set several issues aside. First, although medical age assessments are relevant in different legal contexts, including e.g. in criminal justice [15], we concentrated exclusively on age assessments in the asylum process.

Second, we did not evaluate the scientific evidence for different medical methods that can be used for age assessment. Nor did we take a position on the level of scientific evidence that is required for any such method to be used for this purpose. These issues were important for the revision of the National Board of Health and Welfare’s recommendations. However, they were addressed by internal experts (in the case of radiological methods) and by the Swedish Agency for Health Technology Assessment and Assessment of Social Services (in the case of non-radiological methods) and thus did not form part of the ethical analysis as such.

Third, we did not take positions on migration legislation or migration policy in general. Specifically, we distinguished the question of what an ethically defensible asylum legislation should be like from the question of what an ethically defensible use of age assessments under current legislation should be like, and focused on the latter question. That being said, we noted that different stances on age assessment could reflect different underlying views on migration and that this is one reason why such assessments are controversial. For instance, somebody who holds that the current asylum legislation is too restrictive may object to age assessments on the grounds that they will sometimes result in denial of asylum to adults who would have been granted asylum under more generous legislation (see the “Professional values and roles” section). However, while we recognised the possibility of such an objection, we set it aside and took current Swedish asylum legislation as given for the purposes of our analysis.

The ethical foundations of our analysis were, first, the general ethos of the UN Convention on the Rights of the Child, which assumes a higher degree of vulnerability in children and, therefore, a greater need for protection and, second, the ethical norms codified in Swedish healthcare legislation.

Our analysis proceeded from the framework for systematic identification of ethical issues of healthcare technologies developed by Heintz et al. [16]. The discussion in the remainder of this paper follows the structure of that framework, with some adaptations.

## Ethical analysis

### Goal and effect of the intervention

The use or introduction of any healthcare-related intervention or method is only justifiable if some ethically appropriate goal is thereby furthered. Therefore, the first step when ethically analysing such an intervention or method is to determine whether this is the case. Normally, the goal in question is assumed to be the health or health-related quality of life of patients in the relevant population. Age assessments serve a markedly different set of goals. From a societal point of view, the main goal is to distinguish between individuals who are and who are not entitled to the advantages that are associated with being under 18 years old in the asylum process. Moreover, this should be accomplished in a way that fulfils the basic principles of equal treatment and rule of law that apply to public authorities in general. This requires that the method of assessment is accurate, with as few false positives (individuals incorrectly classified as older than 18) and false negatives (individuals incorrectly classified as younger than 18) as possible. Another important societal goal is to protect children at housing arrangements for asylum-seeking minors who may risk abuse if adults are placed among them.

From the point of view of individual asylum seekers, the main goal is to gain access to the advantages to which children are entitled. Another possible goal is to help their family members secure a residence permit through family reunification. We note that there is no systematic difference between individuals above and below the age of 18 in regard to these two goals. Any asylum seeker, irrespective of actual age, stands to benefit from being considered a child. There is thus a potential conflict between the main societal goal of age assessment and individual asylum seekers’ interest in regard to the procedure.

If one accepts the distinction drawn between children and adults in Sweden’s and many other countries’ asylum laws (as well as in European regulation and the UN Convention on the Rights of the Child), as we do, it follows that the main societal goal is ethically legitimate. Moreover, if one accepts this distinction and the differential treatment it implies, it also appears that one must accept that some method for differentiating children from adults is needed in unclear cases.

It might be argued that these considerations do not support age assessments, since the concern that underlies the legal distinction between children and adults is not with age as such but with protecting individuals who are especially vulnerable. From this perspective, the 18-year age limit is a proxy for a more important distinction between individuals who are and who are not in need of special protection. Moreover, it is an imperfect proxy since some individuals over the age of 18 suffer from vulnerabilities characteristic of children and vice versa. Migration authorities should therefore, the argument goes, assess asylum seekers’ need for protection directly rather than assessing their chronological age in cases where documentation that reliably certifies their age is lacking [6].

Although we agree that the underlying rationale of the 18-year limit is indeed to single out individuals who need special protection, we believe that there are significant concerns with accomplishing this directly. First, such an approach would treat individuals who have reliable documentation and individuals who lack such documentation unequally. The former would presumably be regarded as having the age that is stated in their documents [6], and would thus lack the opportunity (which is available to the latter) of having their need for protection assessed directly. Second, methods that assess psychological maturity (which are presumably required to determine an individual’s need for protection) are less validated than methods that assess chronological age [5, 7, 14]. This makes such psychological assessment problematic from the point of view of equal treatment and rule of law. For these reasons, assessing chronological age seems to be a preferable approach, even though it targets an imperfect proxy.

However, such an assessment could proceed in different ways. One can distinguish between (at least) the following approaches:

1. An investigator at the Migration Agency asks the asylum seeker about their age and takes their stated age at face value.
2. An investigator at the Migration Agency makes an estimation based on the individual’s appearance and behaviour.
3. Medical methods are used with assistance from medical professionals.
4. Psychosocial methods are used with assistance from professional psychologists, physicians, social workers, etc.
5. Some combination of 3 and 4.

An advantage of 1 is that it displays trust in individual asylum seekers. A trusting relationship between authorities and the general public could be considered an important feature of a democratic society. On the other hand, there are many other situations where authorities do not take individuals’ statements at face value when conferring benefits on them but require the statements to be supported by appropriate documentation, e.g. when processing applications for sick leave, income support, or admission to higher education. This is not normally seen as unduly distrustful.

A disadvantage of 1 is that asylum seekers have a strong incentive to state that they are younger than 18 even when they are, in fact, older in order to enjoy the advantages to which children are entitled. The accuracy of this approach therefore appears questionable. Moreover, this approach would arguably favour individuals with the ability to present a credible story about themselves over individuals who are less able to do so. This could be regarded as problematic from the point of view of equality and rule of law.

Alternative 2 raises significant concerns about arbitrariness. Investigators at the Migration Agency are likely to differ considerably in how old they estimate a given asylum seeker to be if they only consider the individual’s appearance and behaviour. Conversely, appearance and behaviour are likely to differ considerably between asylum seekers for reasons unrelated to their age, e.g. cultural background, individual life histories, and individual physical variation. This approach therefore seems difficult to combine with due concern for rule of law and equal treatment. Moreover, intuitive age estimations by individual investigators may be biased by racial or other prejudice or by questionable informal organisational norms or practices, such as the “culture of disbelief” described by Crawley [21] and Aynsley-Green et al. [5] in regard to the British Home Office.

For these reasons, alternatives 3, 4, and 5 seem clearly preferable. However, since the Swedish Agency for Health Technology Assessment and Assessment of Social Services reviewed the literature on psychosocial methods of age assessment in parallel with our analysis and found little evidence in support of them [17], we decided to set 4 and 5 aside.

Regarding alternative 3, the National Board of Health and Welfare reviewed the literature on radiological methods and concluded that magnetic resonance imaging (MRI) of the upper part of the knee appeared to be the most precise method, with a risk of misclassifying children as adults of 3% for males and 7% for females, and that MRI of other parts of the knee and foot showed promise [13]. However, they also proposed a future study to confirm these findings, a study that was subsequently commissioned by the Swedish government [18]. Based on this assessment and in view of the ethical importance of accuracy (see above), our analysis mainly concerned these methods. This will also be the focus in the remainder of this paper.

By concentrating on MRI methods, we could set aside the ethical concerns about radiation risks that have been widely discussed in relation to age assessment using X-ray technology [5, 8, 19] since MRI methods do not use radiation. However, it might be proposed that undergoing an MRI scan could be a frightening experience, especially for individuals suffering from previous psychological trauma, since such scans are commonly loud and require the subject’s entire body to be enclosed by a narrow tube. This concern should be alleviated by the fact that the specific equipment proposed for age assessments in the Swedish context only encloses the targeted body part and emits relatively little noise [13]. Nonetheless, it is possible that individuals with previous experience of abuse from authorities or healthcare professionals (which does not seem unlikely in the case of refugees) may find even such an exam frightening or otherwise unpleasant. Such possible effects need to be taken into account and minimised when implementing age assessments.

### Knowledge gaps

Several authors emphasise that it is impossible to arrive at an exact age for the asylum seeker and that every assessment will involve some degree of inaccuracy [5–7, 14]. Here, it is important that the level of inaccuracy is transparently presented in the report to the Migration Agency, in a way that is understandable also for people without medical expertise. A problem raised in the Swedish public debate is that investigators at the Migration Agency might not be skilled enough in scientific methodology to grasp what the inaccuracy implies, even if it is transparently presented. This problem needs to be taken into account and, if needed, addressed, e.g. by appropriate education.

Considerable uncertainty about an individual’s age can be handled in different ways. Authorities can prefer to (1) err on the side of treating the person as a child (i.e. rather judging him/her to be under 18 than over 18) or, conversely, to (2) err on the side of treating the person as an adult (i.e. rather judging him/her to be over 18). Given the strong ethical norm about greater need for protection for children and in agreement with Directive 2013/32/EU Art. 25(5), we recommend the first approach. This can be related to the degree of uncertainty such that the greater this degree, the stronger the reason to apply this approach [7].

However, although we find this approach to be generally preferable, we note that it is not ethically unproblematic. First, it increases the likelihood of placing adults in housing arrangements intended for children, which may entail risks for these children. Second, in a political climate where authorities seek to keep the number of people granted asylum below a certain figure, it may mean denying asylum to potential applicants with more urgent needs. Hence, such an approach might require enhanced security measures at housing arrangements, but also that authorities consider the possible opportunity cost for other asylum seekers.

### Equality and justice

In a society, we generally find different ethical norms related to equality and justice:

1. Norms about equal treatment (implied by rule of law) and non-discrimination, according to which society should treat similar cases equally [7].
2. Norms about fair distribution of resources. In Swedish healthcare, it is assumed that resources should be distributed according to the principle of need balanced against cost-effectiveness [20], whereas Swedish migration legislation implies that asylum seekers under 18 have the right to a greater share of resources.

As we noted above (see the “Goal and effect of the intervention” section), the first set of norms support an accurate age assessment. However, there is a concern about the scope of applicability of the proposed method. The National Board of Health and Welfare found that the knee joint is sufficiently matured to deliver reliable data on a person’s age in only 42% of 18 year-olds (and 62/71% of 19/20 year-olds) [13]. This suggests that a significant proportion of asylum seekers cannot be assessed with the most accurate method, which is troubling from the point of view of equal treatment and rule of law. Therefore the ambition must be to lower this proportion and/or develop alternative approaches for assessing their age with comparable accuracy.

Another concern is that the risk of misclassification with this method differs between males (3%) and females (7%). From the perspective of equal treatment it is problematic with a method that systematically disadvantages women in this way. Hence, another ambition must be to (as far as possible) lower the risk of misclassifying women.

Given present and probably also future knowledge gaps, no system for age assessment will be able to completely fulfil strict norms about equal treatment. We have suggested that in cases of considerable uncertainty, the person should be assumed rather as to be under than over 18. Moreover, as noted above (see the “Goal and effect of the intervention” section), a system that does not use medical methods risks becoming even less equal with a large degree of arbitrariness, especially if something like a “culture of disbelief” [5, 21] prevails at the Migration Agency.

Another question related to equal treatment concerns the potential long-term effects of age assessments on already existing discriminatory attitudes towards asylum seekers or certain ethnic groups. We address this in the “Long-term effects” section.

Regarding the second set of norms, in Sweden, asylum seekers under 18 have the right to a greater share of resources. Since societal resources are limited, there is an opportunity cost to someone getting such a share without being entitled to it. Here, accurate age assessment can ensure that resources only befall those who have a legitimate claim on them, which is an advantage from the point of view of distributive fairness.

An important question from a healthcare perspective is to what extent age assessments should be allowed to affect healthcare resources. While budgets for migration and healthcare are separate, such assessments require a limited non-monetary resource mainly available in the healthcare sector, namely medical expertise [5, 7]. Age assessments will therefore have an opportunity cost within healthcare, unless the relevant expertise is obtained elsewhere.

### Autonomy

A general ethical and legal requirement on any medical intervention (and many societal interventions) is that the intervention should be preceded by the patient’s valid informed consent. The validity of consent is typically taken to hinge on (at least) three criteria. The person must (1) receive and understand information about the intervention and its effects, (2) be competent to provide a valid consent, and (3) consent voluntarily [22]. In the literature on age assessment, doubts about informed consent have been raised with respect to each of these criteria. Several authors question whether the first criterion is fulfilled given language barriers, difficulties in understanding medical procedures, and lack of information [5, 14, 19]. They also question whether the second criterion is met given asylum seekers’ young age, immaturity, and experienced trauma [5, 14, 19]. Thevissen et al. assume young asylum seekers to be incompetent and suggest that a legal guardian makes decisions on their behalf [7]. The voluntariness is questioned by several writers [6, 11, 14] since a refusal to undergo an age assessment may reduce the chances of an approval on the asylum application.

Let us consider the three criteria applied to the Swedish situation. In Swedish healthcare, children are expected and legally allowed (given a normal trajectory of development and maturity) to participate in decision about their care from an early age and, when in their teens, to increasingly make their own decisions about healthcare. Therefore, systematically assuming asylum seekers under the age of 18 to be incompetent is not in line with existing practice. Obviously, the information provided should be adapted to the individual’s circumstances, including language, (im)maturity, cultural background, and other factors that may constitute barriers for understanding. However, we see no principled reason why the persons in question could not be both competent and able to receive and understand information. It is worth remembering that they have proved capable overcome considerable challenges to get to the point of seeking asylum, which suggests a high degree of independence and autonomy. Still, there must be safeguards in place to ensure that the asylum seeker understands what an age assessment involves and the implications of doing such an assessment. Here, a legally appointed representative could be of assistance.

Authors who raise doubts about voluntariness appear to have in mind a situation where the age assessment is performed on the initiative of the migration authorities, and the asylum seeker can refuse [6]. In such a situation, a refusal might be interpreted to imply that the person has something to hide and bias authorities negatively towards that person. In Swedish praxis, however, an asylum seeker whose age is in doubt is informed about the possibility to request an age assessment, but the initiative then rests with this person. The Migration Agency is not itself involved in performing the actual assessment, and in line with established rules of free sifting of evidence in asylum decisions [23], the applicant may choose not to present its results to the Agency in support of the application. This approach seems preferable from the point of view of voluntariness.

However, although Swedish praxis helps protect voluntariness in a formal sense (i.e. nobody forces or overtly pressures applicants to request an age assessment), it might be argued that it fails to fully protect voluntariness in a more substantial sense. If an applicant whose minority status is questioned chooses not to present evidence that supports their stated age, they will normally be assumed to be over 18. Moreover, in many cases, the only evidence an applicant could produce is the result of a medical age assessment. In such circumstances, applicants appear to have two options: either request an age assessment, which would increase their chances of getting their asylum application approved, or refuse such an assessment, which would entail an increased likelihood of being sent back to difficult circumstances in their home or transit country. It might be argued that the choice of the former option would not be fully voluntary since the only other option is highly unattractive. In other words, the voluntariness of asylum seekers’ consent could be questioned on the grounds that they have “no choice”, i.e. no viable alternative.

Whether or not decisions made in this sort of situation are genuinely voluntary is a matter of longstanding debate in philosophical ethics [24, 25], and we cannot settle the issue here. We note, however, that the decision whether or not to undergo an age assessment does not alone determine an applicant’s prospects of being granted asylum. Requesting an assessment is no guarantee that the application will be granted even if the assessment indicates that the applicant is a minor. Conversely, as per Directive 2013/32/EU Art. 25(5), “the decision to reject an application for international protection by an unaccompanied minor who refused to undergo a medical examination shall not be based solely on that refusal”. Thus, the situation of choice may not be quite as stark, and the threat to substantial voluntariness not quite as severe, as presented above.

We also note that there are other contexts where people are required to provide information or agree to investigations in order to receive substantial benefits from public authorities. For example, a person who wants to obtain paid sick leave in Sweden must consent to healthcare professionals’ sharing information with the relevant authorities. She/he can refuse, but must then also to give up the financial support. Now, although this situation resembles that of asylum seekers, it is admittedly not fully comparable with it. More is generally at stake for asylum seekers and their degree of voluntariness is therefore arguably lower.

It has been argued that there is a further problem with voluntariness if the process for age assessment is perceived as derogatory or implies risks for the person [5]. When it comes to the suggested MRI assessments, we do not see any such risks. However, the investigation requires the person to be completely still when the scans are taken, which presupposes consent or a much larger intervention (like sedation). If the MRI scan is combined with other investigations, the risk might increase. Examining somebody’s genitalia or uncovering body parts the person prefers to remain covered for religious or cultural reasons might here be considered especially problematic [5].

To sum up, given the strong norm about informed consent in the Swedish healthcare and social security system and even if the request of an age assessment cannot be considered fully voluntary in a substantial sense, we find it advisable that the Migration Agency is not directly involved in carrying out the procedure, that it is performed on the initiative of the asylum seeker and that she/he retains the right to decide whether or not to present its results. These measures are important to protect formal voluntariness. Considering the interests at stake, we cannot see that more coercive measures are warranted.

A further autonomy-related question is how the results of the assessment should be treated. In order to make an autonomous decision about whether to present the results to the Migration Agency, it is important that the asylum seeker receives transparent and understandable information about what they show (and do not show) and how accurate the assessment is. Moreover, a legally appointed guardian and the legal representative of the asylum seeker have important guiding roles in regard to that decision.

A related question is how to handle incidental findings of relevance to the person’s health that may be encountered during the investigation. If these findings require healthcare, the person should be informed about this and referred to the relevant healthcare facility. In Sweden, asylum seekers under 18 have full access to healthcare while those over 18 have more limited access. It seems reasonable that all applicants in such a situation are granted full access, given that their age is unknown and that children’s interests are considered to be especially important.

### Privacy

Here, we can distinguish between physical and information-related privacy. Focusing on the methods recommended by the National Board of Health and Welfare, we note that the foot and the knee are not normally considered especially sensitive from a physical privacy perspective. However, even less sensitive body parts might be problematic to investigate if there is no relevant reason for the investigation. Often a health-related objective is such a reason. In this situation, by contrast, asylum seekers might find that substantiating their age in order to strengthen their application is a sufficient reason to outweigh the intrusion. However, some applicants might hold religious or cultural beliefs according to which the foot and the knee are sensitive from a privacy perspective, perhaps especially if exposed to members of the opposite sex. Here, it seems important to be flexible enough to adapt the investigating professional accordingly [5, 7].

Regarding information-related privacy, age assessments collect health-related data, which, in the Swedish context, are considered sensitive information falling under the Patient Data Act (2008:355) and the Swedish Personal Information Act (1998:204). The results of the assessment should therefore be protected accordingly.

It is important to contrast the suggested MRI assessments with alternative methods of age assessment in regard to privacy. Investigation of genitalia to assess sexual maturity clearly appears more intrusive from a physical privacy perspective [5, 7] whereas thorough interviews about background, life experiences, etc., can be viewed as equally or more intrusive from an information-related privacy perspective.

### Professional values and roles

As already mentioned, medical age assessments have been criticised by paediatricians and other affected healthcare professionals. We identified three main objections in the literature. The first one is that available methods for age assessment are insufficiently evidence based and/or insufficiently accurate [11, 12]. Since it was not our task to review the scientific evidence regarding such methods, comprehensively assessing this objection was beyond the scope of our analysis. However, we noted that the objection could potentially be significant. Although the National Board of Health and Welfare estimated that MRI of the knee correctly classifies children as children in 93/97% of cases (for a selected group), they also highlighted a need for more evidence [13]. Should their estimation prove incorrect, concerns about accuracy may well tell against age assessment using this method. Such concerns may also be warranted if other, less accurate methods are used.

The second objection is that age assessments do not aim to promote patients’ health and therefore should not be the concern of healthcare professionals [14]. In response, we noted that the healthcare system is already tasked with a range of other procedures (e.g. elective abortions) and services (e.g. issuing sick notes) that are not strictly speaking related to patients’ health without therefore being considered ethically questionable.

However, according to another version of the objection, age assessments violate the basic healthcare ethical norms of beneficence and/or non-maleficence. Healthcare professionals are supposed to do good to and not harm their patients, which may appear difficult to reconcile with (in some cases) contributing to their not being granted asylum [14]. Here, we noted that, in the context of the Swedish asylum process, an age assessment does, in fact, appear to potentially benefit the individual undergoing it. Asylum seekers may request an age assessment when the Migration Agency questions their stated age and the onus is on them to provide evidence for their statement. In such a situation, individuals who are, in fact, younger than 18 seem to benefit from the opportunity to support their case with the result of an age assessment, whereas older individuals can choose to decline an assessment without decreasing their chance of being granted asylum (provided, as argued above, that the request for an age assessment is informed and voluntary, that the method used is accurate, and that a refusal per se does not negatively affect the asylum decision). This does not exclude that age assessments may fail to benefit applicants in the asylum process in other countries, in which case the objection appears more forceful.

The third objection targets the criteria for granting asylum that age assessments partially serve to uphold. According to this objection, the current asylum legislation is unjustifiably restrictive, and age assessments are ethically questionable because they may contribute to denying asylum to individuals who would have been granted asylum under a less restrictive legislation. Carefully assessing this objection was beyond the scope of our analysis since we assumed that current Swedish asylum legislation is ethically defensible. However, we noted that this starting assumption could itself be subject to ethical scrutiny. Should a more generous asylum legislation be found to be ethically required, this objection would be worth taking seriously as long as the current legislation remains in place. It should be noted, however, that the problem of making sound age assessments will remain under any asylum legislation (no matter how generous) that distinguishes between adults and children in order to assure a higher level of support and opportunities for the latter.

### Long-term effects

There are several potential long-term effects of age assessments worth considering. The first is the impact of age assessments on the general public’s views on migration. Such assessments, especially if widely or frequently practiced, could be interpreted as suggesting that asylum seekers as a group, or more generally the ethnic groups to which they belong, are not to be trusted. This could exacerbate already existing discriminatory societal attitudes towards these groups. Although such a potential effect is worth taking seriously, its likelihood and magnitude are difficult to accurately predict. We offer two preliminary remarks for consideration. First, the possible message that asylum seekers are not trustworthy would be an effect not only of medical age assessments specifically but also (and perhaps primarily) of the general requirement that applicants whose stated age and grounds for asylum are questioned provide evidence or support. Second, it is possible that an accurate and legally robust age assessment process would contribute to reducing rather than exacerbating negative attitudes towards asylum seekers by disarming the suspicion that they gain advantages illegitimately. However, such a positive effect seems more likely in a system where everyone whose stated age is questioned is assessed than in a voluntary system where some choose not to be assessed. The latter system seems to leave more room for public suspicion concerning asylum seekers’ trustworthiness. Nonetheless, such a possible but unsubstantiated positive effect does not, at this point, alter our judgement that age assessments should be voluntary.

Another potential long-term effect is the impact of age assessments on asylum seekers’ motivation to state an incorrect age to gain advantages in the asylum process. The extent to which such behaviour is discouraged is likely to depend on whether age assessments are obligatory and how accurate they are. In general, it is not unlikely that administrative changes affect the information provided by asylum seekers given the strong incentive to get asylum [7].

A third potential long-term effect is the impact on the role of healthcare professionals of being requested to take part in what they might view as non-healthcare interventions. Opportunity costs aside (see the “Equality and justice” section), this might result in moral conflicts and moral stress, especially if professionals see age assessments as incompatible with their general ethos of helping persons or as part of a repressive control system. The potential for such conflicts should perhaps not be exaggerated given that, when assisting with age assessments, healthcare professionals do not seem to be playing the role of healthcare providers per se but rather of expert witnesses in the context of a legal/administrative procedure. In any case, a voluntary system, where asylum seekers themselves request age assessments in order to strengthen their position vis-à-vis authorities, seems less problematic than a mandatory one in this regard. The problem could, of course, also be circumvented by handling age assessments completely outside of the healthcare system, which, again, would require alternative ways of obtaining the relevant competence.

## Summary and conclusions

This paper has provided a simplified and somewhat amended account of the main findings in our recent report on the ethical aspects of medical age assessment of unaccompanied minors who seek asylum in Sweden [4]. Whereas the report discussed a wide variety of aspects, some of which are specific to the Swedish context, this paper has focused on the issues we found most salient from an international perspective.

Our main conclusions are the following:

- As long as one accepts that children ought to enjoy special rights and a certain degree of priority in the asylum process, some way of distinguishing between asylum seekers above and below the age of 18 is needed.
- From the point of view of equal treatment and rule of law, it is essential that the methods used for age assessments have a high degree of accuracy.
- Any uncertainty concerning an assessed individual’s age should be transparently reported to the Migration Agency.
- The Migration Agency’s competence to understand the significance and implications of such uncertainty should be ascertained, if needed by appropriate education.
- In cases of considerable uncertainty, the individual should be regarded as under 18 as it is worse to deny a child its rights that to mistakenly assign those rights to an adult.
- In order to protect formal voluntariness, the decision whether to request an age assessment and whether to present its results to the Migration Agency should rest with the individual asylum seeker. Moreover, the Agency should not be involved in performing the actual procedure.
- To respect the asylum seeker’s autonomy, the age assessment must not be performed without their voluntary informed consent.
- The asylum seeker’s physical and information-related integrity should be respected when age assessments are performed.

Although we have identified several legitimate ethical concerns regarding medical age assessments in the asylum process, we believe that most of them can be addressed in a system where the assessment is sufficiently accurate and adequate safeguards are in place. However, we have also highlighted two issues that would remain in such a system and that require further study and discussion. The first is whether asylum seekers’ consent to the procedure can be considered genuinely voluntary given the potentially dire consequences of declining. The second is whether age assessments will exacerbate or diminish negative public attitudes towards asylum seekers or discriminatory/racist views more generally.

In closing, we wish to suggest that perhaps the most fundamental and challenging ethical question in regard to the asylum process is not whether and how to perform age assessments, but rather what a society’s basic criteria for granting asylum should be. However, we also hope to have made clear that this question demands separate analysis.
